# Non-specialist delivery of the WHO Caregiver Skills Training Programme for children with developmental disabilities: Stakeholder perspectives about acceptability and feasibility in rural Ethiopia

**DOI:** 10.1177/13623613231162155

**Published:** 2023-05-16

**Authors:** Tigist Zerihun, Mersha Kinfe, Kamrun Nahar Koly, Rehana Abdurahman, Fikirte Girma, WHO Team, Charlotte Hanlon, Petrus J de Vries, Rosa A Hoekstra

**Affiliations:** 1 University of Cape Town, South Africa; 2 Saint Paul’s Hospital Millennium Medical College, Ethiopia; 3 Addis Ababa University, Ethiopia; 4 King’s College London, UK; 5 International Centre for Diarrhoeal Disease Research, Bangladesh; 6 Yekatit 12 Hospital Medical College, Ethiopia; 7 World Health Organization, Switzerland

**Keywords:** caregivers, developmental disabilities, non-specialist facilitators, World Health Organization caregiver skills training

## Abstract

Autism and other developmental disabilities are common in low- and middle-income countries. The World Health Organization developed the caregiver skills training programme to address the needs of families having children with developmental disabilities globally. This study explored the acceptability and feasibility of the caregiver skills training programme facilitated by non-specialists in rural Ethiopia, where contextual factors such as poverty, stigma and low literacy may affect training delivery. In-depth interviews were conducted with caregivers (n = 19) and four focus groups with non-specialist facilitators (n = 8) in two rural pilot tests of the caregiver skills training programme. Results suggested that participants experienced the caregiver skills training programme as acceptable and relevant for their context but suggested some modifications to improve the programme. Caregivers’ accounts showed that facilitation by non-specialists was acceptable; facilitators emphasised the importance of supervision throughout the programme. Participants indicated that the home visit and group training modalities were acceptable and feasible. Facilitators indicated that some caregiver skills training programme topics were difficult to explain to caregivers; the concept of formalised play between caregiver and child was particularly foreign to caregivers. Lack of available toys made it difficult to practise some of the caregiver skills programme training exercises. These findings may have relevance to non-specialist delivery of the caregiver skills programme training and other parent-mediated interventions in low-resource contexts.

**Lay abstract:**

Children with developmental disabilities including autism who live in low- and middle-income countries have very limited access to care and intervention. The World Health Organization initiated the caregiver skills training programme to support families with children with developmental disabilities. In Ethiopia, contextual factors such as poverty, low literacy and stigma may affect the success of the programme. In this study, we aimed to find out if the caregiver skills training programme is feasible to deliver in rural Ethiopia and acceptable to caregivers and programme facilitators. We trained non-specialist providers to facilitate the programme. Caregivers and non-specialist facilitators were asked about their experiences in interviews and group discussions. Caregivers found the programme relevant to their lives and reported benefits of participation. Facilitators highlighted the skills they had acquired but also emphasised the importance of support from supervisors during the programme. They described that some caregiver skills training programme topics were difficult to teach caregivers. In particular, the idea of play between caregiver and child was unfamiliar to many caregivers. Lack of available toys made it difficult to practise some of the caregiver skills training programme exercises. Participants indicated that the home visits and group training programme components of the caregiver skills training were acceptable and feasible, but there were some practical barriers, such as transportation issues and lack of time for homework practice. These findings may have importance to non-specialist delivery of the caregiver skills training programme in other low-income countries.

## Introduction

Autism and other developmental disabilities (DD) are priority conditions for intervention efforts around the globe, particularly in low- and middle-income countries (LMIC), where 95% of the global population of children with DD live (Olusanya et al., 2018). The number of young children with DD has increased by 71% in sub-Saharan Africa (SSA) in the last 25 years (Olusanya et al., 2018), and Ethiopia was identified by Olusanya and colleagues (2018) as being in the top 10 SSA countries in terms of number of children with DD.

Children with DD typically need services that address behavioural, developmental and educational challenges. However, access to, and availability of, services to support their needs is highly limited in SSA (de Vries, 2016; Franz et al., 2017; Lord et al., 2022) and in Ethiopia, in particular (Tekola et al., 2016). Moreover, SSA countries have an extreme shortage of human resources for DD and mental health services in general (Galvin & Byansi, 2020). For instance, Ethiopia has four child psychiatrist for a population of 115 million and only nine other professionals who are specialised in child mental health (World Health Organization (WHO), 2020). To address this service gap in LMIC and other low-resource contexts, the WHO and collaborators developed an open-access training programme to strengthen caregiving skills for families of children with DD (Salomone et al., 2019), referred to as the caregiver skills training (CST) programme. The CST is a manualised, relatively low-intensity training programme for caregivers of children with DD between the ages of 2 and 9 years that was designed to be feasible for wide-scale implementation (Salomone et al., 2019). The programme consists of 12 sessions in total: 9 sessions delivered to groups of caregivers plus 3 home visits to the individual family homes. The programme content is based on principles of social communication interventions, developmental science, applied behaviour analysis, positive parenting and self-care methods (Salomone et al., 2019) and as such is aligned with naturalistic developmental behavioural interventions (Schreibman et al., 2015). The CST programme targets caregiver skills to enhance communication, increase shared engagement, improve adaptive behavioural skills and reduce and prevent challenging behaviour in their children (Salomone et al., 2018; Szlamka et al., 2022).

The WHO CST programme was largely considered acceptable, relevant and feasible in urban settings in Italy, Hong Kong and India (Salomone et al., 2022; Sengupta et al., 2021; Wong et al., 2022). Its positive impact has also been reported from an urban setting in Serbia (Glumbic et al., 2022). Studies about the feasibility and acceptability of WHO CST programme are scarce in SSA. The WHO CST programme has been adapted for use in a rural low-resource setting in South Africa (Schlebusch et al., 2020). Implementation in this setting of a short caregiver well-being module complementary to the main CST programme was found to be feasible and acceptable (Schlebusch et al., 2022). Other CST adaptations and evaluations are underway in Zambia and Kenya (Salomone et al., 2019).

In Ethiopia, the CST programme underwent an initial contextual adaptation and was tested in a pre-pilot conducted with mental health specialists as facilitators in an urban clinical setting. Stakeholders in that study reported that the CST programme was feasible and acceptable (Tekola, Girma, et al., 2020). In addition, this study identified the clear need for this kind of service in a context where no other services were available for caregivers of children with DD (Tekola, Kinfe, et al., 2020).

Even though these were encouraging findings, most people in Ethiopia live in rural communities and cannot access centralised services run by specialists. A promising approach to scale up human resources is task-sharing (Divan et al., 2021). Using the task-sharing approach, the intervention facilitator role, traditionally assigned to a specialist, is shared with a trained non-specialist, who is responsible for the main intervention facilitation under continuous specialist supervision. Non-specialist facilitators who speak the local languages, understand the context and are part of the local community, are ideally placed to deliver a community-based intervention. However, at present, the evidence of acceptability and feasibility of non-specialist delivery of CST or similar programmes for autism and other DD in LMIC is still limited (Divan et al., 2021). This study aimed to assess the acceptability and feasibility of the CST programme delivered by non-specialist facilitators in a rural Ethiopian setting from the perspectives of both caregivers and facilitators.

## Methods

### Study design

The study used a qualitative design comprising in-depth interviews and focus group discussions.

### Setting

The study was conducted in two districts in the Gurage zone, Southern Nations, Nationalities and People’s Region of Ethiopia. Butajira and Sodo are predominantly rural districts located 135 and 99 km, respectively, south of the capital city Addis Ababa. Both districts have populations with low socioeconomic status and livelihoods that rely mainly on agriculture (Dendir & Simane, 2019).

### Participants

The study included two participant groups from two different pilot tests of the CST programme: (1) caregivers of children with DD and (2) non-specialist CST facilitators. Table 1 shows details of the two data sets, participants and samples included in the study. Data set I was collected as part of a pre-pilot of the CST, when the CST programme was first tested in a rural setting and facilitated by non-specialists. The pre-pilot was also the first test of our training for non-specialist facilitators. This data set comprised two CST groups, each attended by 10 caregivers, with no comparison group. Data set II was a pilot randomised wait-list controlled field trial study (Trial ID: PACTR201812802696820) in rural Sodo and Butajira involving 66 families, with half of the families enrolled in four CST groups (the intervention arm), and half of the families in four wait-list control groups (the control arm). Caregiver participants for this nested qualitative study were drawn from the intervention arm.

**Table 1.** Data sets and sample size.

Data set	Description	Participants
Data set I	Pre-pilot in a community setting in rural Sodo district involving 20 families	In-depth interviews with caregivers (N = 9) Focus group discussions with non-specialist facilitators (N = 7)
Data set II	Pilot randomised wait-list controlled field trial study in rural Sodo and Butajira involving 66 families	In-depth interviews with caregivers (N = 10) Focus group discussion with non-specialist facilitators (N = 8)

#### Caregivers

The CST programme included a total of 86 caregivers as participants (pre-pilot (N = 20) and pilot randomised controlled trial (RCT; N = 66)). Criteria for inclusion of caregivers were (1) having a long-term caring responsibility for a child aged 2–9 years with DD, preferably as the primary carer; (2) living within easy travelling distance of a training site; (3) caregiver ability to attend the full number of sessions; (4) having sufficient contact time during the week with the child with DD (seeing the child at least 5 days a week on average) to do homework exercises; (5) ability to speak Amharic, the official language in the study site; (6) being 18 years of age or older; and (8) willingness to provide written informed consent. Exclusion criteria included (1) children who were acutely disturbed and/or in need of specialist medical attention and (2) children with severe visual or hearing impairments (since many of the CST strategies will not be helpful for children with these impairments. For this qualitative study, caregivers were purposively selected from the larger group of CST participants based on caregiver gender, the religious or educational background of the caregiver and diagnosis of the child, ensuring representation of a range of educational and religious backgrounds and different DD diagnoses and inclusion of fathers as well as mothers.

#### Non-specialist facilitators

**Figure 1.**
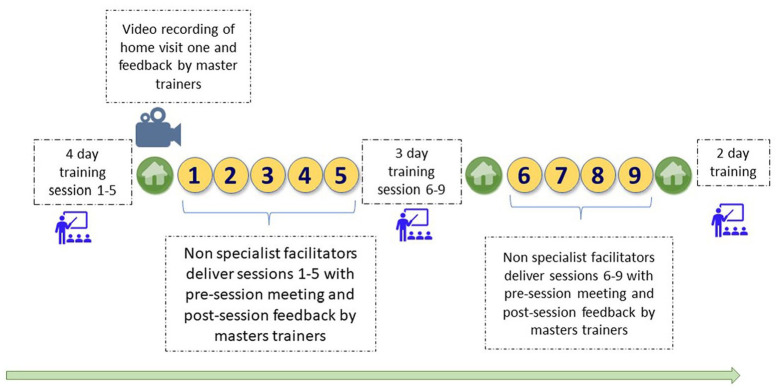
Overview of training, supervision and delivery of CST using non-specialist facilitators in Ethiopia (CST sessions delivered to caregiver groups in yellow, three home visits to families in green).

All non-specialist facilitators were selected from the district where they worked and comprised community health workers (health extension workers), primary health care workers (nurses and health officers) or teachers. Non-specialist facilitators received 4 days of initial training focusing on CST group sessions 1–5 and home visit 1 and then assisted the delivery of these sessions to caregivers in practice. Two facilitators led the group sessions: one lead (who facilitated the training session) and one assistant (who assisted the lead facilitator with role plays, demonstrations and timekeeping). Each lead CST facilitator had previous experience of at least one full round of CST delivery as assistant facilitator; most assistant facilitators were newly trained and thus new to CST delivery. Master trainers reviewed a video recording of the home visits and goal setting sheets and provided comprehensive feedback to the facilitators to improve goal-setting and facilitation skills. A further 3 days of training focused on CST group sessions 6–9 and home visits 2 and 3. This was followed by the completion of assisting the delivery of the full CST programme by the non-specialists under the supervision of the master trainers. Finally, the non-specialists completed two further days of training focusing on troubleshooting, a review of the complete programme, and role-plays conducted to practice skills that focused on leading rather than assisting the facilitation of the programme (see Figure 1). Following completion of a full round of CST delivery, some assistant facilitators moved on to become lead facilitators. Thirteen non-specialists started the training initially, eight of whom completed the full training cycle. Five dropped out due to maternity leave or competing responsibilities.

### Supervision and support

Facilitators received ongoing supervision and support from CST master trainers throughout the programme. Supervisory meetings took place before each session CST group session and lasted 30 min to an hour. During these meetings, any challenges encountered by non-specialists during preparation were discussed. In addition, brief feedback sessions were arranged for the facilitators after they completed the delivery of each CST group session.

During the pre-pilot, supervisors attended all nine weekly CST group sessions, whereas, in the pilot RCT, supervisors attended only six out of nine weekly CST sessions (sessions 1, 2, 3, 6, 8 and 9) with the same pre-session supervisory and post-session feedback meetings.

### Research procedures and data collection

In-depth interviews and focus group discussions were conducted with caregivers and facilitators, respectively. Demographic data were collected on age, gender, marital status and educational level. The topic guides focused on programme content, experience and remaining needs from both the caregivers’ and facilitators’ perspective. All non-specialist facilitators who facilitated the pilot studies were invited to participate in focus group discussions. The interviews and focus groups of participants in data set I were collected following the completion of CST group session 6. Data set II was collected after completion of the full CST programme. All qualitative interviews and focus group discussions were conducted in Amharic (the official language of Ethiopia) by independent researchers who did not have any role in the CST programme facilitation and were audio-recorded. Informed consent was obtained from all individual participants included in the study.

### Data analysis

Audio recordings were transcribed verbatim in Amharic and translated into English by bilingual Amharic/English researchers from Addis Ababa University. The transcripts were checked against the audio files by the first author who led the analysis. The data sets were analysed together, with an effort made to compare the caregiver and facilitator perspectives. The framework approach was used for analysis. This approach encompasses five interconnected stages: (1) familiarisation, (2) identifying a thematic framework, (3) indexing, (4) charting, reviewing themes and (5) mapping and interpretation (Bryman & Burgess, 1994). After familiarisation with the interview transcript data and reviewing the relevant literature, and informed by previous research experience in this context, including the urban pilot study of CST (Tekola, Girma, et al., 2020) and the broader experience of co-investigators in implementing mental health interventions in the study site, the research team developed a specific framework (see Table 2). Sample interviews were coded using the framework, and discussions were held between authors T.Z., C.H., P.J.d.V. and R.A.H. on sample coding, resulting in revisions to the framework. After the final review of the framework, all the data were coded using the final framework by the first author of this study.

**Table 2.** Framework developed for data analysis.

Themes	Subthemes
1. What: Programme content	Acceptability and relevance
	Complexity of concepts
	Contextual appropriateness
	Overall programme perception
2. By whom: Programme facilitation	Acceptability of non-specialist facilitators to caregivers
	Support and supervision
3. How: CST training approach and delivery	Feasibility of CST in the local context
	Programme delivery structure

CST: caregiver skills training.

The data from the pre-pilot study were independently coded by another researcher (K.N.K.). K.N.K. reviewed the first author’s framework coding, and K.N.K. and T.Z. met to discuss and resolve any discrepancies in codes.

During the coding, the researchers kept in mind that the participants were from different data sets collected from different study phases. The research team also considered differences in facilitator levels of exposure to CST (the facilitators had had more experience delivering the CST in the pilot RCT phase than in the pre-pilot study). NVivo (Version 12) software was used to facilitate the analysis (QSR, 2018).

### Positionality

We acknowledge and reflect on our insider positionality and potential contextual biases as researchers based in an LMIC context. The primary author who performed the analysis was a CST master trainer and supervisor of the non-specialist facilitators with first-hand experience of the context; this might affect the interpretation of the data. Furthermore, she worked in child and adolescent mental health services as a psychiatrist in a resource-limited setting with two other co-authors. All the authors have worked in, and conducted research in, low-resource settings and subscribe to the principle of developing initiatives to scale up mental health services to reach as many people as possible given the limited resources available, using task-sharing as an example. Thus, we tend to gravitate towards a model that uses non-specialists and may be tolerant of potential disadvantages of task-sharing. The perspective is distinct from a specialist working in a high-income context with relatively better resources.

### Ethical approval

This study was approved by the Institutional Review Board of the College of Health Sciences of Addis Ababa University (#062/16/Psy), the Psychiatry, Nursing and Midwifery subcommittee of King’s College London’s College Research Ethics Committee (#RESCM-17/18-3489) and by the Health Research Ethics committee of the Faculty of Health Sciences, University of Cape Town (HREC 476/2021).

### Community involvement

This study benefited from the long-term involvement of a project advisory board, a group of stakeholders in Addis Ababa including parents of children with DD, founders of special schools for children with DD, representatives of local and international non-governmental organisations, and experts in child mental health and education. This project advisory board advised the research team on important research questions, methods and measures, and directly informed the adaptation of the CST programme, as previously outlined in the study by Tekola, Girma, et al. (2020). This study was unable to solicit the views of Ethiopian people with autism or other DD themselves. In Ethiopia, autism tends to only be identified and diagnosed in children who also have intellectual disability and are typically minimally verbal.

## Results

### Demographic information

#### Caregivers

Nineteen caregivers participated in in-depth interviews. Their age range was from 30 to 70 years, and the majority (15/19) were women. More than half of the participants (10/19) could not read or write and only one caregiver had education beyond high school Table 3.

**Table 3.** Demographic information of caregivers and children.

Caregiver study Code	Main diagnosis of child	Child age (years)	Child sex	Caregiver relationship to the child	Caregiver educational level
C01^a^	Autism + ID	4	Male	Father	Secondary
C02^a^	ID	9	Male	Mother	Primary
C03^a^	ID	7	Male	Mother	Primary
C04^a^	Autism	7	Male	Mother	No education
C05^a^	Autism + ID	9	Male	Mother	No education
C06^a^	ID	8	Female	Father	Tertiary
C07^a^	ID	6	Female	Mother	Basic literacy
C08^a^	Autism	5	Male	Mother	Secondary
C09^a^	ID	7	Female	Mother	Secondary
C10	GDD	3	Male	Mother	No education
C11	ID	2	Female	Mother	No education
C12	ID	4	Male	Mother	No education
C13	GDD	3	Male	Mother	No education
C14	ID	5	Female	Mother	Secondary
C15	ID	3	Female	Mother	Secondary
C16	ID	5	Female	Mother	No education
C17	ID	10	Male	Mother	No education
C18	Autism	6	Male	Father	No education
C19	ID + CP	12	Female	Father	No education

ID: intellectual disability; GDD: global developmental delay; CP: cerebral palsy.

a Participants from the pre-pilot study (data set I); C10–C19 were participants in the pilot RCT study (data set II).

#### Non-specialist facilitators

A total of eight facilitators (four male and four female) participated in three focus group discussions and one group interview. Four facilitators were primary or junior high school teachers, and four were primary healthcare workers. To avoid unequal power dynamics during the focus groups, each focus group contained only lead facilitators who were the main trainers or assistant facilitators who assisted the group sessions. After the pre-pilot five assistant facilitators participated in a focus group, two lead facilitators participated in a group interview. Following the pilot RCT, four assistant facilitators were part of one focus group discussion and four lead facilitators were part of a second focus group. Seven facilitators were interviewed twice, once after the pre-pilot and once after the pilot RCT of Ethiopian CST study.

### Framework analysis findings

In this section, the findings are presented under the three themes identified for framework analysis and outlined in Table 2: (1) What: CST programme content, (2) By whom: programme facilitation and (3) How: CST training approach and delivery. Illustrative quotes are marked as C (for caregivers), LF (for lead facilitators) and AF (for assistant facilitators).

#### Theme 1: CST programme content

The programme content theme included four subthemes addressing acceptability and relevance of CST for the context, the complexity of concepts, contextual appropriateness and overall programme perception.

### Acceptability and relevance

Several caregivers described the programme as relevant for their circumstances because it helped them address their children’s needs. They reported that the programme dealt with issues specifically caused by the child’s problem, such as challenging behaviour and poor caregiver well-being. Caregivers often noted that they found the CST relevant for their children and themselves:

In all sincerity, CST is very valuable . . . I left all my work to come here so that I wouldn’t miss it. (C03)

Another participant also emphasised that she had wished it was available in her area before joining the CST programme:

It [CST] was something that I had always wanted; it is just something that is available in larger cities only. (C01)

Facilitators also described the programme as relevant to the community and themselves, even for those with physical comorbidities:

The programme is excellent. It has even shown me things clearly on problems I did not know before as a professional. (AF-02)

The training is also relevant for those with physical disabilities. Although the physical disability cannot be reversed, the cognitive delay can be improved by this training. We witnessed that. (AF-06)

Participants remarked that the programme was acceptable and appropriate for their culture. Caregivers specifically mentioned that the content was highly relevant to their lives:

The feeling, the stress, crying, fear in the story [Caregiver Story, caregiver vignettes presented throughout the training as a way to introduce a topic and promote identification], all are related to me. (C16)

Facilitators also emphasised that the training content was acceptable overall. However, they commented on the hesitation of some caregivers to accept some content or activities, such as playing with the children. The concept of a caregiver playing with their child, rather than children playing among themselves, was unfamiliar for many caregivers. For instance, one lead facilitator said,

Sitting on the floor and playing with the child took them a long time to accept and practice that. (LF-01)

Participants indicated their interest in future use. Caregivers indicated that they would like to continue the programme. Facilitators also expressed interest in participating in a similar programme in the future:

It is beneficial, I have enjoyed it a lot, and I wish this could continue in the future. (C06)

Since I have seen good outcomes while doing this job, I would like to continue this programme and help them. (LF-01)

### Complexity of concepts

Caregivers indicated that most of the concepts in the training manual were clear to them:

For me the lessons are very clear, there isn’t a part that you would hate or want to take out, I think it is easy to understand. (C02)

In contrast to the caregiver reports, non-specialist facilitators indicated that some concepts and techniques were difficult to understand and to explain to caregivers and required extra practice and time. For example, the concept that children may engage in different levels of play, from simple play to advanced pretend play, was difficult for both facilitators and caregivers:

It is difficult for them to identify and increase the level of their children’s play. Some of the parents do not even know their children’s level of play. (LF-02)

Facilitators commented that despite the apparent interest in practising the home routine activities in the training manual, some parents struggled to understand how to set small clear steps in their daily routines:

Sometimes activities [home routines] like setting teachable small steps of washing the dishes is complex for the participant. (AF-02)

Facilitators found the need to tailor some content to meet the participants’ different literacy levels. They reported that the concepts were time-consuming to explain to parents and for themselves to understand:

We were working in a community where most of them are not educated. Due to the individual illiteracy, they were not able to understand easily what we try to train them. (AF-01)

### Contextual appropriateness

Under this subtheme, we discuss the findings on clarity of the illustrations used in the CST materials, availability of toys and homework practice.

#### Clarity of illustrations

Facilitators emphasised the overall value of the illustrations in the caregiver manual and how it helped to train caregivers with no education:

Most of the time, caregivers understand the illustrations. I think a caregivers’ booklet should have illustrations that account for more than 75 per cent of its content. (AF-08)

In addition, the illustrations helped the facilitators to elaborate when the caregivers experienced difficulty understanding the discussion. However, they commented that some illustrations from the participant booklet were unclear and incompatible within their rural setting:

The illustrations did not represent the context. Most people do not have tap water access, and in the manual, it says ‘by opening the water tap’, and it would have been nice if that could have been substituted with a ‘water jug’; therefore, I think the illustrations should be modified so that they reflect the actual living conditions of society. (AF-01)

#### Availability of toys

Facilitators indicated that some toys or materials listed in the booklet were unavailable to families. For instance, the facilitators demonstrate by using a toy cup for the child and the family. However, caregivers did not have the toys to practice and were afraid to practice with an actual cup in case it got broken:

The child shows an interest in playing, but the materials are unavailable. The families might not have any toys for the child to play with. This problem was seen in many households. (AF-07)

Another facilitator gave an example of how challenging it was for caregivers to get toys:

For example, one of the activities we showed them can be performed with puppets ordered on the shelf, but caregivers won’t get them. (AF-06)

#### Homework

Caregivers reported that the homework allowed them to understand the material and opportunity to practice with other family members. Despite these reported benefits, there were some challenges, for instance, a lack of time to complete homework:

I could not practice [the homework] as we planned. We have a high workload which never ends. (C19)

Facilitators also described how difficult it was for caregivers to complete their homework practice between sessions due to busy home schedules:

When they [caregivers] were asked as to why they did not do it [homework] they said we were busy. (AF-02)

### Overall programme perception

Caregivers emphasised how the programme helped them to understand their child’s problems and improve their skills to support their child. Facilitators highlighted having acquired new knowledge and skills relating to DD.

Caregivers commented on specific behavioural changes based on the CST strategies in their children, such as their child’s increased capacity to communicate, to help themselves, increased engagement and an increased capacity to control their own behaviour:

My son couldn’t ask for water and food before the training. Based on my training, I taught him how to ask for what he needs. It helped both my son and myself. (C15)

Another participant said,

During the training, every single thing was new to me. I had no idea what I was doing, and I gained a lot of new knowledge and skills. (C19)

Facilitators also described how their understanding improved after taking part in CST and how they benefited from the CST:

The training taught me more about neurodevelopmental disorders and delays than I had learned while at university. (AF-03)

#### Theme 2: Programme facilitation

The programme facilitation theme included two subthemes: addressing the acceptability of having non-specialists facilitate the programme and the ongoing support and supervision of facilitators by master trainers.

### Acceptability of non-specialist facilitators to caregivers

Caregivers described that facilitation by non-specialists was acceptable. Indeed, they expressed that using local non-specialists was essential to ensure that caregivers who were more comfortable speaking local languages could participate. Caregivers described the facilitators as being knowledgeable about the content, skills and resources needed to support their children. They also appreciated the facilitators’ patience, compassion and the way they interacted with the caregiver and family:

There is nothing difficult to understand and even for those who can’t read they are teaching us very well. (C06)

Another caregiver described how the facilitators took time to explain the content:

The trainers were excellent. I don’t even have words to explain. There were different behaviours [in the booklet] they took the time to explain to us. They made us feel free to tell what was on our minds. (C16)

One of the caregivers also stated that she was happy with how the non-specialist facilitators handled difficult issues during CST group sessions:

The facilitators are extremely patient; there are some participants who don’t understand things easily, and they ask unrelated questions. Trainers explain things in more detail and gently encourage caregivers to return to the topic of discussion. That is very good behaviour of the trainers, and I look at it with much respect. (C14)

### Support and supervision

The facilitators emphasised the importance of supervision in the programme. They particularly valued feedback received from the supervisors and highlighted the need for discussion with the supervisor at the end of each session. Having a supervisor who they could consult, together with additional teaching from the supervisor in response to their emerging needs, was viewed as essential by the non-specialist facilitators:

It is very important that we have people who supervise us. For instance, if there is something we are unclear about, we talk to them at the end. They asked if there were any challenges we encountered while delivering the training. Therefore, it is essential to have those who supervise us. (LF-02)

Generally, facilitators identified that they struggled to comprehend some of the CST concepts in their early days of training, but their understanding improved after further practice and training. One of the facilitators said they were well equipped for the second round of CST groups after participating in the pre-pilot:

Due to the fact that the challenges we faced during the first round of training did not exist during the second round and that we were more familiar with the training materials allowed for more time to prepare for the training. (LF-01)

#### Theme 3: CST training approach and delivery

Results under this theme focused on feasibility of CST in the local context, including perspectives on training modalities, and on the structure of programme delivery, including training.

### Feasibility of CST in the local context

Participants indicated that the training modalities, including three individual home visits and nine group sessions to caregivers, were acceptable and feasible in the local context:

Both sections, the support they have given us during the home visit and the programme here is very helpful and convenient; it is not something that has any problem. (C05)

However, some caregivers reported barriers to attending the group session on time, including transportation, family responsibilities and childcare:

I arrived late, I was sweaty, I have walked to the hospital, which is far from my home on foot. Since there is no *bajaj* [a three-wheel motorbike], the moment I arrive here, I am sweaty, and I miss the section about behaviour. (C01)

Despite some challenges, the facilitators also agreed that CST was doable in their context. For example, one lead facilitator said,

Most of the time, or I should say all of the time, we were able to conduct the training without any problems. (LF-02)

### Programme delivery structure

#### Group sessions

All caregivers described the group setting as beneficial and stated that they enjoyed the social support. Multiple caregivers highlighted a benefit of group sessions:

Having met other caregivers made me realise that I’m not the only one dealing with this issue. (C07)

Despite this benefit, one caregiver said she had difficulty adjusting to being in a group in early training days. She indicated the non-specialist facilitators helped her cope with it:

I was scared of learning with others and talking about things in front of people. They [the facilitators] helped me to be able to sit in the group setting while I took the training. (C15)

Facilitators also emphasised the benefits of the group sessions. An assistant facilitator said:

By participating in the group, they had an opportunity to share their experiences. (AF-06)

#### Home visits

Caregivers described that the home visit helped them to understand what they can do for their child and allowed demonstration of CST strategies in the natural environment of the child and caregivers:

I have learned a lot from the lesson they gave us by coming to our home. I am happy about the home visit. I have observed several changes in my child because of their lesson by coming up to our home. (C07)

Facilitators described that the home visit was acceptable, helpful and that caregivers were accommodating. The facilitators observed that the caregivers felt more comfortable expressing themselves in their homes compared to in group settings:

Parents might have questions they were not comfortable asking during group sessions. When we visit their home, we will give them the chance to ask those questions and also review for them some of the topics they had difficulty understanding. (LF-02)

Despite the positive perception of home visits by facilitators, they raised the challenges of travelling long distances to families’ homes:

In this town, the houses are scattered and far from each other, so we have to walk long distances to do home visits. (AF-01)

## Discussion

This study explored the acceptability and feasibility of non-specialist delivery of the WHO CST programme from the perspectives of caregivers and non-specialist facilitators who participated in the programme in rural Ethiopia. Findings showed that the non-specialist-delivered CST programme, adapted for use in Ethiopia (Tekola, Girma, et al., 2020), was valued due to perceived relevance and usefulness, and was culturally acceptable and largely feasible, but needed several further contextual adaptations to be appropriate for a rural Ethiopian context.

All participants reported that the training had given them new insights, with caregivers reporting that the CST helped to increase understanding of their child’s condition and improved their capacity to manage their child. Facilitators commented on how caregivers noticed a positive change in their children throughout their participation in the training. Caregivers commented on specific behavioural changes based on the CST strategies in their children, such as the increased capacity to communicate. These descriptions of perceived benefits are in line with reports from participants in CST when delivered by specialists in a clinical setting in Addis Ababa (Tekola, Girma, et al., 2020) and in Italy (Salomone et al., 2022). Similarly, a study from an urban setting in India reported the acceptability and feasibility of non-specialist-delivered WHO CST (Sengupta et al., 2021). Unlike these previous CST studies, our study was done in a rural low-income context where the majority of caregivers had limited education. It is encouraging that the programme is also perceived as beneficial by this participant group.

Other existing reports also have noted the benefits of parenting programmes in improving parental knowledge and skills understanding and managing challenging behaviour in children (Karst & Van Hecke, 2012). Several studies also indicated that exchanging ideas with others and feeling safe to talk was important for parenting programme participants (Cutress & Muncer, 2014; O’Donovan et al., 2019; Todd et al., 2010; Toure et al., 2020). In addition, our study indicated that some caregivers in a rural Ethiopian context may need extra encouragement and support to feel comfortable in group sessions.

Generally, home visits and group sessions were deemed to be acceptable and feasible in rural Ethiopia, but with some practical challenges reported by participants, such as travel time and the busy schedule of caregivers. These findings on challenges and barriers to implementing the WHO CST programme are consistent with previous CST studies in Ethiopia and Italy (Salomone et al., 2022; Tekola, Girma, et al., 2020). Other studies of parenting programmes have also identified similar barriers (Koerting et al., 2013; Mytton et al., 2014; Pickard et al., 2019; Straiton et al., 2021; Tekola, Girma, et al., 2020). Interestingly, our finding that caregivers struggled to fit in the time to practice at home contrasted with findings in Italy, where participation in home practice was good. In that study, they found that ability to keeping the child engaged and additional support to help caregivers were factors that contributed to home practice adherence (Salomone et al., 2022).

Some of the challenges reported in this study were partially reported by the previous Ethiopian CST study and not at all by studies from other settings, such as the lack of toys in the homes (Salomone et al., 2022; Tekola, Girma, et al., 2020). Ethiopian children play with ordinary objects found within their household environment (Berhanu, 2006), rather than with toys. The initial adaptation to the Ethiopian context (outlined in the study by Tekola, Girma, et al., 2020) already included more emphasis on household items and items in the environment such as sticks and leaves than the original WHO CST materials. This study suggests that these adaptations are even more important in the rural Ethiopian context, where resources are scarce and children are very unlikely to have toys.

A more fundamental challenge was the culturally alien concept of a caregiver playing with the child. In Ethiopia, play between parent and child is uncommon and not typically thought of as an opportunity for parent–child interaction or learning (Metaferia et al., 2021). However, parents spend time with their children in household activities such as feeding and bathing, and these activities (also included in the CST) come more naturally to Ethiopian parents than the play activities. There are a number of sayings and proverbs that illustrate this cultural norm, such as, ‘ከልጅ አትጫወት ይወጋሃል በእንጨት’ [‘Do not play with children, otherwise they will pierce you with a stick’]. The findings of this study are in line with those of the WHO CST in Italy where the cultural beliefs of traditional parent–child interaction do not facilitate application of the concept of teaching children through play (Salomone, 2022). In this study, the facilitators reported that caregivers did try to play, even when initially hesitant, but a more culturally consonant approach may be to adapt CST to focus on playful interactions during the daily routines such as bathing, cleaning, meal preparation and washing clothes, where caregiver and child naturally interact.

Delivering the CST programme in a setting with low literacy, low awareness, no other child mental health services, high levels of stigma (Tekola, Kinfe, et al., 2020), common beliefs that a child with DD is unable to learn and high expectations of a cure, brings extra challenges (Tekola, Kinfe, et al., 2020). In this study, fewer than half of the participants were literate, and only one caregiver had education beyond high school. This is reflective of the rural context in which the study was conducted and is representative for much of Ethiopia outside urban areas. In these contexts, CST programmes require an increased focus on psychoeducation and simplification of participant booklets, including more illustrations and discussing concepts orally rather than in writing. In addition, non-specialist facilitators need access to ongoing support from supervisors, to allow them to grow in their role of making complex content accessible to caregivers. This task-sharing approach to intervention facilitation can be a promising model to scale up services in a rural context.

### Strengths and limitations of the study

We acknowledge various limitations affecting the scope and breadth of this study and the analysis of the findings. First, the possibility of socially desirable responses is a real challenge in most low-resource contexts, including in Ethiopia. We acknowledge that caregiver participants may have been more likely to evaluate any programme positively when no other services were available. However, to reduce this possibility, we employed strategies, such as including an experienced qualitative study researcher independent of the CST facilitating team as interviewer, who established a good rapport and encouraged participants to give their honest opinions about the programme. Second, caregivers were interviewed at different stages of completion of the CST, that is, after CST group session 6 in the pre-pilot study and after completing the full nine programme sessions in pilot RCT. This was considered during the analysis, but may have affected some of their responses. The facilitators were interviewed at two different time points, with more work experience facilitating the CST after the second focus group discussion. This could also be considered a strength, as this study was able to capture their perspectives during different points in their training journey. Finally, the primary author of this research who analysed the data was also the supervisor and the master trainer of the programme. This may, therefore, have biased her interpretation of data. To mitigate against bias in data analysis, all interviews and focus group discussions were led by researchers uninvolved in the CST facilitation and supervision, and we included an external researcher (K.N.K.) who had not participated in programme adaptation and implementation in the analysis as a second coder.

A strength of this study is the unique perspective provided on implementing an intervention for families with children with DD in rural very low-income contexts, where the majority of caregivers have low levels of literacy. Autism intervention research is heavily skewed towards western, high-income countries (de Vries, 2016; Lord et al., 2022) with over-representation of parents with higher socioeconomic status and education. However, the majority of the global population of children with autism and other DD live in LMIC (Olusanya et al., 2018), and many of the caregivers caring for these children have limited levels of education. This study provides unique insights on how to support families in these contexts of very high unmet need. These results have relevance to other low-income contexts too. A recent study in stakeholders involved in adapting or implementing the CST programme across five continents and different income settings reinforced the importance of contextual adaptations of interventions, highlighting, in particular, the importance of socioeconomic context (Szlamka et al., 2022).

## Conclusion

In this study, we found that the adapted and non-specialist delivered WHO CST programme was greatly valued as it was perceived to be relevant and useful, culturally acceptable and generally feasible, but it required several contextual adaptations, as well as a simplification of programme content and delivery, to be appropriate for a rural Ethiopian context. Regular supervision of non-specialist providers was identified as an essential ingredient These findings suggest that the CST programme has the potential to be scaled up for families of children with DD in Ethiopia and other low-resource contexts. We hope that these results will be useful in informing the implementation of the CST programme and other parent-mediated interventions in LMIC.
